# Neuroprotective Effect of Magnoflorine Through Inhibition of Caspase‐3‐Mediated Neuronal Apoptosis: In Silico to In Vitro Validation

**DOI:** 10.1002/jbt.71171

**Published:** 2026-11-07

**Authors:** Randeep Singh, Sahil Arora, Rimple Kaur, Ali Gharib Gharib, Vikash Prashar, Tania Arora, Arti Sharma, Balachandar Vellingiri, Jyoti Parkash

**Affiliations:** ^1^ Department of Zoology, School of Basic Sciences Central University of Punjab Bathinda Punjab India; ^2^ Department of Pharmaceutical Sciences and Natural Products, School of Health Sciences Central University of Punjab Bathinda Punjab India; ^3^ Department of Computational Sciences, School of Basic Sciences Central University of Punjab Bathinda Punjab India; ^4^ Department of Allied Health Sciences, School of Allied and Healthcare Sciences Lovely Professional University Phagwara Punjab India

**Keywords:** Caspase‐3, Kainic acid, Magnoflorine, molecular docking, neuroprotective

## Abstract

Neurodegeneration is a progressive biological phenomenon that is significantly driven by neuronal apoptosis. The current study aimed to investigate the neuroprotective potential of Magnoflorine, a natural alkaloid, by inhibiting Caspase‐3‐mediated neuronal apoptosis induced by Kainic acid. The caspase‐3‐inhibiting property of Magnoflorine was initially predicted using in silico methods such as molecular docking, MMGBSA analysis, and molecular dynamics (MD) simulations, taking Kainic acid as the reference neurotoxic ligand. Molecular docking and MMGBSA analysis showed that Magnoflorine has a higher binding affinity for caspase‐3 than that of Kainic acid, which predicts the potential of Magnoflorine to inhibit caspase‐3 expression. MD simulations further highlighted this potential through the greater stability of the Caspase‐3‐Magnoflorine complex than the Caspase‐3‐Kainic acid complex over 200 ns of simulations. It was finally evaluated through multiple in vitro techniques such as the MTT cell viability assay, LDH cytotoxicity assay, Annexin V‐PI apoptosis assay, and immunocytochemistry performed on the retinoic acid‐differentiated SH‐SY5Y cells. MTT and LDH assays revealed that Magnoflorine significantly improved the viability of differentiated SH‐SY5Y cells by reducing Kainic acid–induced cell death and cytotoxicity. The apoptosis assay also showed that Magnoflorine significantly reduced the Kainic acid‐induced apoptosis in differentiated SH‐SY5Y cells. Inhibition of Caspase‐3 by Magnoflorine became evident during immunocytochemistry through the reversal of the Kainic acid‐induced increase in Caspase‐3 expression in differentiated SH‐SY5Y cells, as observed from immunofluorescence intensity. The present study suggests that Magnoflorine is emerging as a valuable therapeutic agent for neurodegeneration by alleviating neuronal apoptosis, which requires further validation through in vivo studies.

## Graphical abstract

The in silico analyses provided a basis for the neuroprotective potential of magnoflorine through its anti‐apoptotic effect by inhibiting the overexpression of caspase‐3 induced by Kainic acid. Our further investigations through in vitro analyses confirmed the anti‐apoptotic property of magnoflorine against the Kainic acid‐induced neuronal apoptosis in retinoic acid‐differentiated SH‐SY5Y cells.

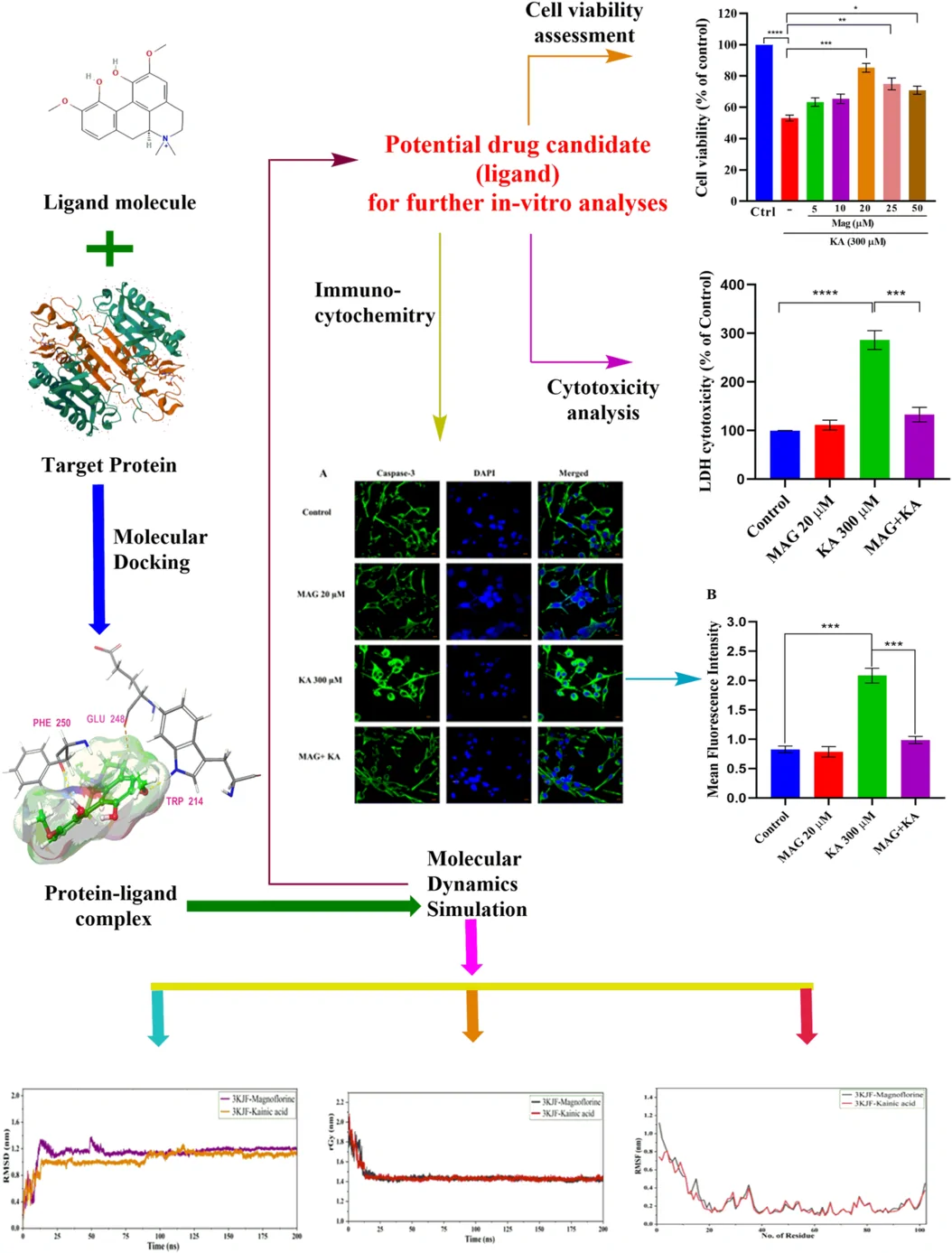

## 1 Introduction

Neurodegeneration progressively deteriorates normal brain functioning, which leads to memory loss, cognitive decline, depression, and many other abnormalities. It is the typical process of several neurodegenerative diseases, including Alzheimer's disease (AD), Parkinson's disease (PD), Huntington's disease (HD), Amyotrophic lateral sclerosis (ALS), spinocerebellar ataxia (SCA), and some neurological disorders like Epilepsy [1, 2]. Neurodegenerative diseases are generally treated with synthetic drugs that aim at slowing down the progression of neurodegeneration and, hence, improving the quality of life to some extent.

Synthetic drugs targeting neurodegenerative diseases take a longer time to develop, and their success rate is dramatically lower (8.2%), with low efficacy in slowing disease progression and significant side effects [3, 4]. Natural products, like phytochemicals, on the other hand, possess many therapeutic properties and fewer side effects, which highlights their use to treat various neurodegenerative diseases. Numerous phytochemicals have been isolated from leaves, roots, and stems of various medicinal plants, and many of them have demonstrated drug‐likeness, as identified and evidenced by their pharmacokinetic analysis. Bioactive phytochemicals undergo a series of investigations before being declared as a drug, including in silico methods such as molecular docking with the biological or therapeutic target, pharmacokinetic analysis, and molecular dynamics (MD) simulations, in vitro and in vivo testing, and extensive clinical trials.

The impact of molecular docking and MD simulations in molecular biology and drug discovery has expanded dramatically in recent years [5]. Phytochemicals often exhibit multiple biological activities due to their complex chemical structures and diverse interactions with biological systems, and evaluating individual activities in a randomized experimental study is a complex and time‐consuming procedure. In this scenario, the use of molecular docking and MD simulations becomes a more suitable approach to initially predict the medicinal strength of any desired phytochemical before conducting a randomized experimental study [6]. It is considered an advanced and cost‐effective technique to avoid the random, practical, or ‘trial‐and‐error’ method of drug screening [7, 8, 9].

In the current study, we investigated and compared the molecular interactions and binding affinities of Magnoflorine (a phytochemical) and Kainic acid (a neurotoxin) with a biological target that plays a crucial role in neurodegeneration and apoptosis, that is, Caspase‐3, using molecular docking, MMGBSA analysis, and MD simulations. Magnoflorine is a widely distributed alkaloid found in numerous genera of flowering plant families, such as Ranunculaceae, Menispermaceae, and Magnoliaceae. It is an active constituent of several commonly used herbal medicines, including Sinomenium acutum, Coptis chinensis, Phellodendron amurense, Berberis kansuensis, and Tinospora cordifolia. Magnoflorine is a quaternary ammonium alkaloid with molecular formula C_20_H_24_NO_4_^+,^ which indicates its high polarity and solubility in water, methanol, and ethanol [10]. It has an aporphine configuration with two hydroxyl (‐OH) groups at positions 1 and 11, two methoxyl (‐OCH_3_) groups at positions 2 and 10, and two methyl groups (‐CH_3_) on the nitrogen atom, which favors its polarity and water solubility. Magnoflorine demonstrated a higher binding affinity than Kainic acid towards Caspase‐3, as evidenced by its more negative docking score and binding energy observed during molecular docking and MMGBSA analysis. It also showed higher stability with Caspase‐3 during MD simulations. Further, we performed in vitro assays such as 3‐(4, 5‐dimethylthiazolyl‐2)−2, 5‐diphenyltetrazolium bromide (MTT) cell viability, lactate dehydrogenase (LDH) cytotoxicity, apoptosis assay, and immunocytochemistry to analyze the neuroprotective effect of magnoflorine against Kainic acid‐induced neuronal apoptosis. Kainic acid is an excitatory neurotoxin that stimulates or hyperexcites neurons at a lower dose [11] while inducing neuronal death via caspase‐3‐mediated apoptosis at a higher dose [12, 13]. Caspase‐3 is a key mediator of apoptosis in neuronal cells [14]. Caspase‐3 is selected as the primary molecular target for investigating neuronal apoptosis observed in various neurodegenerative diseases because it acts as the central executioner caspase in both the intrinsic (mitochondrial) and extrinsic (death receptor) pathways. Its activation represents the “point of no return” in the dismantling of neuronal structures, making it a definitive marker for measuring the extent of apoptosis [15, 16, 17]. Moreover, its inhibition by synthetic compounds, including non‐peptidyl inhibitors like M826 and peptidyl inhibitors such as N‐benzyloxycarbonyl‐Asp(OMe)‐Glu(OMe)‐Val‐Asp(OMe)‐fluoromethyl ketone (Z‐DEVD‐FMK), has proven to be neuroprotective [18, 19]. However, non‐peptidyl inhibitors are widely used due to their better cell permeability, stability, and oral bioavailability in comparison to peptidyl inhibitors, which possess low cell permeability and instability. The alleviation of Caspase‐3‐mediated neuronal death by magnoflorine observed in the present study highlights its neuroprotective potential. Magnoflorine is a non‐peptidyl molecule due to its alkaloidal nature, which makes it a better alternative to the synthetic drugs used to treat various neurodegenerative diseases. Synthetic drugs, hence, need to be replaced by natural medicines that exert substantial therapeutic effects with fewer side effects.

## 2 Materials and Methods

### 2.1 Reagents

Magnoflorine (SMB00377, Sigma‐Aldrich, USA) was dissolved in dimethyl sulfoxide (DMSO, D2650, Sigma‐Aldrich, USA) for use in experiments. Kainic acid (HY‐N2309, MedChem Express, USA) was dissolved in ultrapure water to make the 10 mM stock solution. IC50 value for Kainic acid i.e 300 µM was evaluated using an MTT assay performed in a retinoic acid‐differentiated SH‐SY5Y cell line, as shown in Figure S1 (provided in the supplementary material).

### 2.2 Molecular Docking

We studied the molecular interactions of Caspase‐3 (PDB ID: 3KJF), a protease involved in neuronal apoptosis or neurodegeneration, with ligands such as Magnoflorine and Kainic acid through molecular docking. The two‐dimensional (2D) structures of both ligands were retrieved from the PubChem database (https://pubchem.ncbi.nlm.nih.gov). The PubChem CIDs for Magnoflorine and Kainic acid are 73337 and 10255, respectively. The molecular docking analysis was conducted on these ligands using the Glide module within the Maestro software suite (version 13.5) of Schrödinger (version 2023‐1). The retrieved 2D structures of these ligands were imported into Maestro in sdf format [20]. Ligand preparation was performed with the help of the LigPrep tool to generate 3D structures of ligands from their 2D structures, utilizing the OPLS4 force field, where the ionization states were set at pH: 7.0 ± 2.0. Tautomers were generated for each neutralized or ionized molecule by retaining the specified chiralities, up to 5 per ligand [21].

The chemical structure of the Caspase‐3 protein was obtained from the RCSB Protein Data Bank and imported into Maestro using a protein preparation wizard, followed by its preprocessing or preparation. The preparation of the protein structure involved assigning bond orders, adding missing hydrogens, including zero‐order bonds to metal and disulfide bonds, and removing water molecules beyond 5.00 Å [21]. The active chain, where the co‐crystallized ligand was bound, underwent further refinement, including optimization, removal of remaining water molecules, and restrained minimization [22]. The ligand and protein preparation was followed by the generation of a receptor grid, which defined the active site of the protein, creating a receptor‐grid file. A flexible ligand‐protein docking was performed by docking the prepared ligands into the active site of the prepared protein, utilizing the receptor grid file [21]. The root mean square deviation (RMSD) was computed by applying the extra precision mode to determine the docking scores of ligands and their binding affinities towards Caspase‐3. The molecular interactions are demonstrated in terms of docking scores that indicate the binding affinity of ligands with the target protein. A more negative score corresponds to a stronger binding affinity [23].

### 2.3 MMGBSA Binding Analysis

The MMGBSA (Molecular Mechanics Generalized Born Surface Area) method in Schrödinger involves calculating the binding free energy of ligand‐receptor complexes post‐docking. The procedure begins by performing molecular docking using the Glide module to generate the ligand‐receptor complex. After docking, the Prime MMGBSA module is employed to calculate the binding energy (ΔG bind) using molecular mechanics (MM) for the potential energy of the complex, combined with the Generalized Born (GB) model to estimate the solvation effects [24]. This is followed by calculating the surface area (SA) to account for non‐polar solvation effects. The total binding free energy is derived by considering the difference in the energies of the ligand‐receptor complex, the free ligand, and the receptor. MMGBSA is particularly useful for rescoring docked poses and providing a more accurate assessment of binding affinities [20].

### 2.4 Density Functional Theory (DFT) Optimization

DFT optimization using Schrödinger (version 2023‐1) was carried out to evaluate the electronic structure and stability of magnoflorine. The process began by importing the molecular structure of magnoflorine into the Maestro (version 13.5) interface, followed by performing a geometry optimization using the Jaguar module. The DFT method was selected with an appropriate functional, such as Becke‐3‐parameter Lee‐Yang‐Parr (B3LYP) [25] and a suitable basis set like 6‐31 G for optimization [26]. The optimization minimized the molecule's energy by adjusting bond lengths, angles, and dihedrals. Following this, the energies of the Frontier molecular orbitals, such as the Highest Occupied Molecular Orbital and Lowest Unoccupied Molecular Orbital, along with other molecular properties like dipole moments and total energy, were calculated. The results provide insight into the molecule's electronic distribution, chemical reactivity, and stability [20].

### 2.5 Molecular Dynamics (MD) Simulations

Molecular dynamics (MD) simulations were performed based on docking results, with magnoflorine and caspase‐3 as one complex and kainic acid and caspase‐3 as another complex exhibiting the best binding affinities. Consequently, the two complexes were subjected to MD simulations using GROMACS (version 2020.1) for 200 ns to assess protein stability post‐ligand interaction and potential conformational changes [27, 28]. UCSF Chimera (version 1.17.3) was used to separate the ligand and protein for preprocessing, generate individual topology files, and then combine them to prepare each complex for simulation [29]. The complex topology was generated using the CHARMM27 force field with the TIP3P model to solvate the water box with a minimum distance of 1.5 nm from the surface of the protein [30]. Na^+^ and Cl^‐^ ions were used as counter ions to neutralize the system, followed by energy minimization through the steepest descent algorithm with a maximum of 50,000 steps [31]. Subsequently, NVT equilibration was conducted for 100 ps at 300 K using a V‐rescale thermostat, followed by a 100 ps NPT equilibration, with similar parameters (300 K, 1 bar) applied using a Berendsen barostat, followed by a 200 ns MD simulation [32]. Analyses of Root Mean Square Deviation (RMSD), Root Mean Square Fluctuation (RMSF), and Radius of Gyration (rGy) were conducted to assess stability, compactness, and structural changes.

### 2.6 Cell Culture

The human neuroblastoma cell line SH‐SY5Y (RRID: CVCL_0019) used in this study was purchased from the National Centre for Cell Sciences (NCCS), Pune, India, in 2025. The cell line was authenticated by the supplier (NCCS) using short tandem repeat (STR) profiling and tested negative for mycoplasma contamination during Hoechst staining. SH‐SY5Y cells were cultured at 37°C in a 5% CO_2_ incubator using Dulbecco's Modified Eagle Medium (DMEM, 11965092, Gibco, USA) containing 10% fetal bovine serum (FBS, 10270106, Gibco, USA) and 1% 100X antibiotic‐antimycotic (15240062, Gibco, USA) solution.

### 2.7 Cell Viability Assay

SH‐SY5Y cells were seeded in 96‐well culture plates at a density of 10^4^ cells/ml and incubated overnight at 37°C in an incubator with optimal humidity and 5% CO_2_. The fully grown cells were differentiated with 10 µM retinoic acid (R2625, Sigma‐Aldrich, USA) dissolved in DMEM supplemented with 1% FBS for 3 days according to the refined protocol [33]. The differentiated cells were treated in triplicate (indicating three technical replicates) with various concentrations of Magnoflorine (5, 10, 20, 25, and 50 µM) for 24 h. After 24 h, cells were further treated for the next 24 h with Kainic acid at 300 µM concentration. The Kainic acid‐treated medium was then replaced with 0.5 mg/ml MTT (MB186, HiMedia, INDIA) solution prepared in DMEM, which was kept for 3 h at 37°C in a humidified incubator containing 5% CO_2_. The formazan crystals formed in the wells were dissolved in DMSO, and the absorbance was read at 570 nm with a microplate reader (TECAN Spark, Switzerland). The whole procedure was performed two more times, indicating three independent biological replicates.

### 2.8 Cell Cytotoxicity Assay

Cell cytotoxicity induced by the Kainic acid treatment was evaluated by measuring the LDH released from the damaged cells using a CyQUANT LDH cytotoxicity assay kit (C20300, Invitrogen, USA). SH‐SY5Y Cells were seeded in 96‐well plates at a density of 10^4^ cells/ml and differentiated with 10 µM retinoic acid in DMEM with 1% FBS for 3 days. The differentiated cells were pre‐treated in triplicate with 20 µM Magnoflorine for 24 h before being treated with 300 µM Kainic acid for the next 24 h. One set of triplicate wells was considered the maximum LDH activity controls (MLAC). After 24 h of treatment with Kainic acid, cells labeled as MLAC were lysed with 1X lysis buffer for 45 min at 37°C in a humidified incubator with 5% CO_2_. 50 µL of medium from each well was transferred to new respective wells, and 50 µL of the reaction mixture was then added to each new well. The plate was kept in the dark at room temperature for 30 min. 50 µL of the stop solution was added to each well, and the absorbance was read at 490 nm and 680 nm using a microplate reader (TECAN Spark, Switzerland). Cytotoxicity was measured as the percentage of the control. The whole procedure was performed two more times, indicating three independent biological replicates.

### 2.9 Annexin V‐PI Apoptosis Assay

Apoptosis was evaluated via flow cytometry using an Annexin V‐FITC/PI Apoptosis Kit (V13242, Invitrogen, USA). SH‐SY5Y cells were seeded in a 6‐well plate at a density of 10^5^ cells/mL and differentiated for 3 days with 10 µM retinoic acid prepared in DMEM containing 1% FBS. The differentiated cells were treated in triplicate with 20 µM magnoflorine and left for 24 h, after which 300 µM Kainic acid was added. Kainic acid‐treated cells were incubated for 24 h and then trypsinized. The resulting cell pellet was washed with cold phosphate‐buffered saline (PBS) to eliminate any residual medium or trypsin that could interfere with the fluorescence of the dye. After removing the PBS, the cells were resuspended in 100 µl of 1X Annexin binding buffer to prepare a single‐cell suspension. To this, 5 μL of Annexin‐V FITC and 1 μL of PI (100 μg/mL) were added. The cells were incubated in the dark at room temperature for 15 min to allow proper staining and then placed on ice. Following incubation, the stained cells were analyzed using the Attune NxT (Thermo Scientific, USA) flow cytometer. The percentage of cells in each of the fourquadrants, based on the Annexin‐V FITC and PI staining, was determined to assess the live, necrotic, and apoptotic populations [34]. The whole procedure was performed two more times, indicating three independent biological replicates.

### 2.10 Immunocytochemistry

The neuroprotective effect of magnoflorine against the Kainic acid‐induced neurodegeneration was assessed by analyzing the changes in the expression level of Caspase‐3 through immunocytochemistry. SH‐SY5Y cells were seeded on coverslips kept in 12‐well plates at a density of 10^5^ cells/mL and differentiated for 3 days with 10 µM retinoic acid prepared in DMEM containing 1% FBS. The differentiated cells were treated in triplicate with 20 µM magnoflorine and left for 24 h. After 24 h, 300 µM Kainic acid was given for the next 24 h. The treatment media were removed, and cells were washed twice (5 min each) with ice‐cold 1X PBS. PBS‐washed cells were fixed with a mixture of acetone and methanol (1:1) for 15 min. The fixed cells were washed twice with 1X PBS and permeabilized with 0.3% PBST for 15 min. Cells were again washed twice (5 min each) with 1X PBS, and blocking was done for 30 min with 2% bovine serum albumin (BSA, 199898, MP Biomedicals, USA) solution prepared in 0.3% PBST. Cells were incubated overnight in a humid chamber at 4°C with Caspase‐3 (NB100‐56708SS, Novus Biologicals, USA) at a dilution of 1:500 as a primary antibody. The humid chamber was kept for 1 h at room temperature, and cells were washed thrice (5 min each) with 0.1% PBST before the secondary antibody incubation, that is, anti‐mouse IgG FITC (F0257, Sigma‐Aldrich, USA) at a dilution of 1:500. After the secondary antibody incubation for 2 h, cells were washed thrice (5 min each) with 0.1% PBST and then with 1X PBS twice (5 min each). Cells were incubated with DAPI (D9542, Sigma–Aldrich, USA) solution prepared in 1X PBS for 5–7 min at room temperature and then washed thrice (5 min each) with 0.1% PBST and twice with 1X PBS (5 min each). Coverslips containing the cells were mounted on clean glass slides using 1–2 µL of Mowiol (81381, Sigma–Aldrich, USA), and the images were taken at 60X with a confocal microscope (Olympus F1200, Japan). The images were analyzed with ImageJ software (version 1.53t, NIH, USA) to calculate the mean fluorescence intensity, which indicates the expression level of Caspase‐3. The whole procedure was performed two more times, indicating three independent biological replicates.

### 2.11 Statistical Analysis

Statistical analysis was performed using GraphPad Prism 8 software (GraphPad Software, San Diego, CA, USA). Error bars were presented as the mean ± SEM. The statistical significance between different groups was determined using one‐way ANOVA followed by Tukey's post‐hoc test. *p* < 0.05 was considered statistically significant.

## 3 Results

### 3.1 Molecular Interactions of Caspase‐3 With Magnoflorine and Kainic Acid

The molecular docking results highlight distinct binding affinities and interactions of Magnoflorine and Kainic acid at the active site of Caspase‐3 (PDB ID: 3KJF), providing insights into their predicted interactions with this target protein, that is, Caspase‐3. Magnoflorine showed a more favorable predicted interaction with Caspase‐3 than Kainic acid, which indicates its higher binding affinity towards Caspase‐3, also evidenced by its more negative docking score (kcal/mol) and Glide G score than those of Kainic acid, as shown in Table 1.

**Table 1:** Docking scores (kcal/mol) of Magnoflorine and Kainic acid when docked against the Caspase‐3 (PDB ID: 3KJF) protein.

S. No.	Receptor	Ligand	Docking score (kcal/mol)	Glide G score (kcal/mol)
1	3KJF	Magnoflorine	−4.235	−4.666
2	3KJF	Kainic acid	−4.059	−4.062

*Note:* A more negative score of Magnoflorine indicates its stronger binding affinity towards Caspase‐3 than that of Kainic acid.

Magnoflorine exhibited specific interactions with amino acid residues within the Caspase‐3 binding site. The hydroxy group attached to the tetrahydronaphthalene moiety of magnoflorine formed a hydrogen bond with the PHE250 residue. Moreover, the methoxy group attached to the tetrahydroisoquinolinium moiety of magnoflorine participated in a hydrogen bond interaction with the TRP214 residue. Additionally, the tetrahydropyridinium moiety of magnoflorine formed a hydrogen bond with the GLU248 residue. These interactions indicate the involvement of multiple residues within the Caspase‐3 binding pocket in the predicted binding mode of Magnoflorine.

Kainic acid also exhibited hydrogen‐bond interactions with similar amino acid residues within the Caspase‐3 binding site. The carboxylic acid groups and pyrrolidine moiety of Kainic acid formed hydrogen bonds with SER209, TRP214, and PHE250 residues. Thus, Magnoflorine and Kainic acid shared interactions with TRP214 and PHE250 residues, while their predicted interaction patterns differed with respect to other residues, particularly GLU248 for Magnoflorine and SER209 for Kainic acid. Under the applied docking protocol, these differences in the interaction pattern were accompanied by a more favorable predicted docking pose for Magnoflorine. To further contextualize the predicted binding mode of Magnoflorine, the established non‐peptidyl Caspase‐3 inhibitor M826 was included as a reference comparator. M826 occupied the same Caspase‐3 binding region and displayed interactions within a similar local amino‐acid environment to that observed for Magnoflorine. The comparative docking poses and molecular interactions of Magnoflorine and M826 are presented in the supplementary material (Figure S2). Overall, the docking analysis demonstrated differences in the predicted molecular interaction patterns of Magnoflorine and Kainic acid within the Caspase‐3 binding site, with Magnoflorine showing a more favorable predicted interaction under the applied docking conditions (as shown in Figure 1A,B).

**Figure 1:**
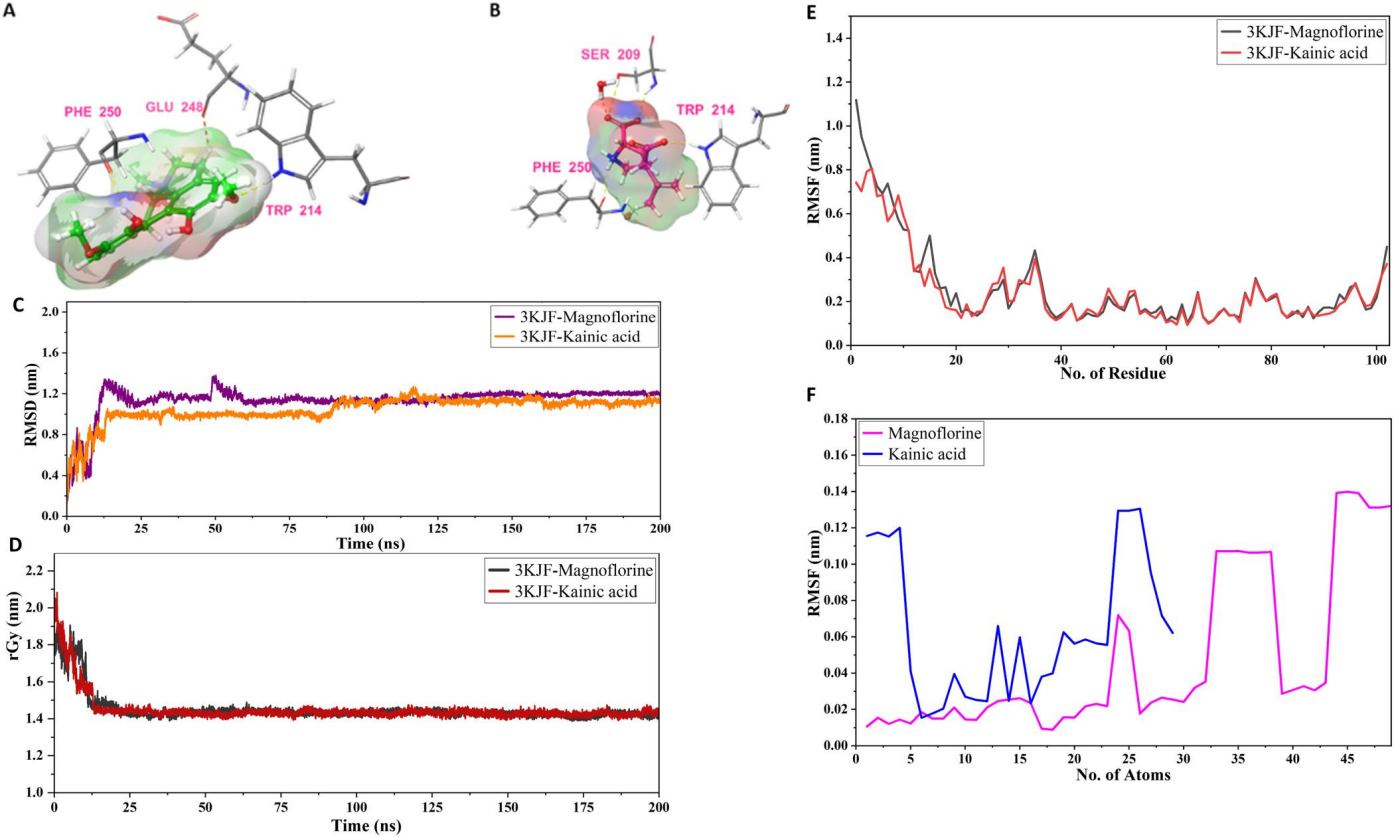
Molecular docking and molecular dynamics (MD) simulation analysis of the Magnoflorine‐Caspase‐3 and Kainic acid‐Caspase‐3 complexes. Three‐dimensional (3D) representation of significant binding interactions of (A) Magnoflorine and (B) Kainic acid at the active site of Caspase‐3 (PDB ID: 3KJF). (C) Root Mean Square Deviation (RMSD) profiles of the Caspase‐3 (3KJF)‐Magnoflorine (Mag) and Caspase‐3 (3KJF)‐Kainic acid (KA) complexes over 200 ns of MD simulation. (D) Radius of Gyration (rGy) values of the Caspase‐3 (3KJF)‐Magnoflorine (Mag) and Caspase‐3 (3KJF)‐Kainic acid (KA) complexes over 200 ns of MD simulation. (E) Root Mean Square Fluctuations (RMSF) plot of the Caspase‐3 (3KJF)‐Magnoflorine (Mag) and Caspase‐3 (3KJF)‐Kainic acid (KA) complexes over 200 ns of MD simulation. (F) RMSF plot of Magnoflorine (Mag) and Kainic acid (KA) over 200 ns of MD simulation.

### 3.2 MMGBSA Analysis

The MMGBSA analysis was carried out to evaluate the binding energies of magnoflorine with the target protein Caspase‐3. When magnoflorine was bound to Caspase‐3, the prime energy and ΔG bind were −3827.6 kcal/mol and −40.63 kcal/mol, respectively. The breakdown showed Coulomb energy of −4.29 kcal/mol, covalent binding energy of 2.79 kcal/mol, hydrogen bond energy of −1.05 kcal/mol, and solvation energy of 6.11 kcal/mol. These results highlight the strong binding affinities of magnoflorine with Caspase‐3, as indicated by their low ΔG bind values. In terms of energy components, Coulomb (electrostatic) interactions were more favorable, contributing significantly to the binding. Covalent interactions were modest, Hydrogen bond contributions were relatively small but present, and Solvation energy (which includes the interaction of the ligand with the solvent) varied. When Kainic acid was bound to Caspase‐3, the prime energy and ΔG bind were −3900 kcal/mol and −26.19 kcal/mol, respectively. The breakdown showed a Coulomb energy of −27.52 kcal/mol, covalent binding energy of 1.31 kcal/mol, hydrogen bond energy of −2.68 kcal/mol, and solvation energy of 34.95 kcal/mol. It was revealed that magnoflorine binds with better affinity to caspase‐3 and has a good binding energy (ΔG bind) compared to kainic acid.

### 3.3 DFT Calculations

DFT analysis of magnoflorine was conducted using Schrödinger to evaluate its electronic properties. The energies of the Highest Occupied Molecular Orbital and the Lowest Unoccupied Molecular Orbital were determined, which are key indicators of a compound's ability to donate or accept electrons, respectively. For magnoflorine, the energy of the Highest Occupied Molecular Orbital was calculated as −0.309 eV, and the Lowest Unoccupied Molecular Orbital as −0.134 eV, resulting in an energy gap (ΔE) of 0.175 eV (as shown in Figure 2). A considerably smaller energy gap suggests higher reactivity and a greater likelihood of participating in chemical reactions, indicating the ease with which the molecule can donate or accept electrons. The Highest Occupied Molecular Orbital—Lowest Unoccupied Molecular Orbital energy gap is a critical factor in determining the chemical reactivity and stability of a compound. A smaller energy gap means the compound can easily transition between electron‐donating and electron‐accepting states, making it more reactive. Magnoflorine shows a smaller energy gap, suggesting it may be more chemically reactive, which could influence its biological activity in a cellular environment.

**Figure 2:**
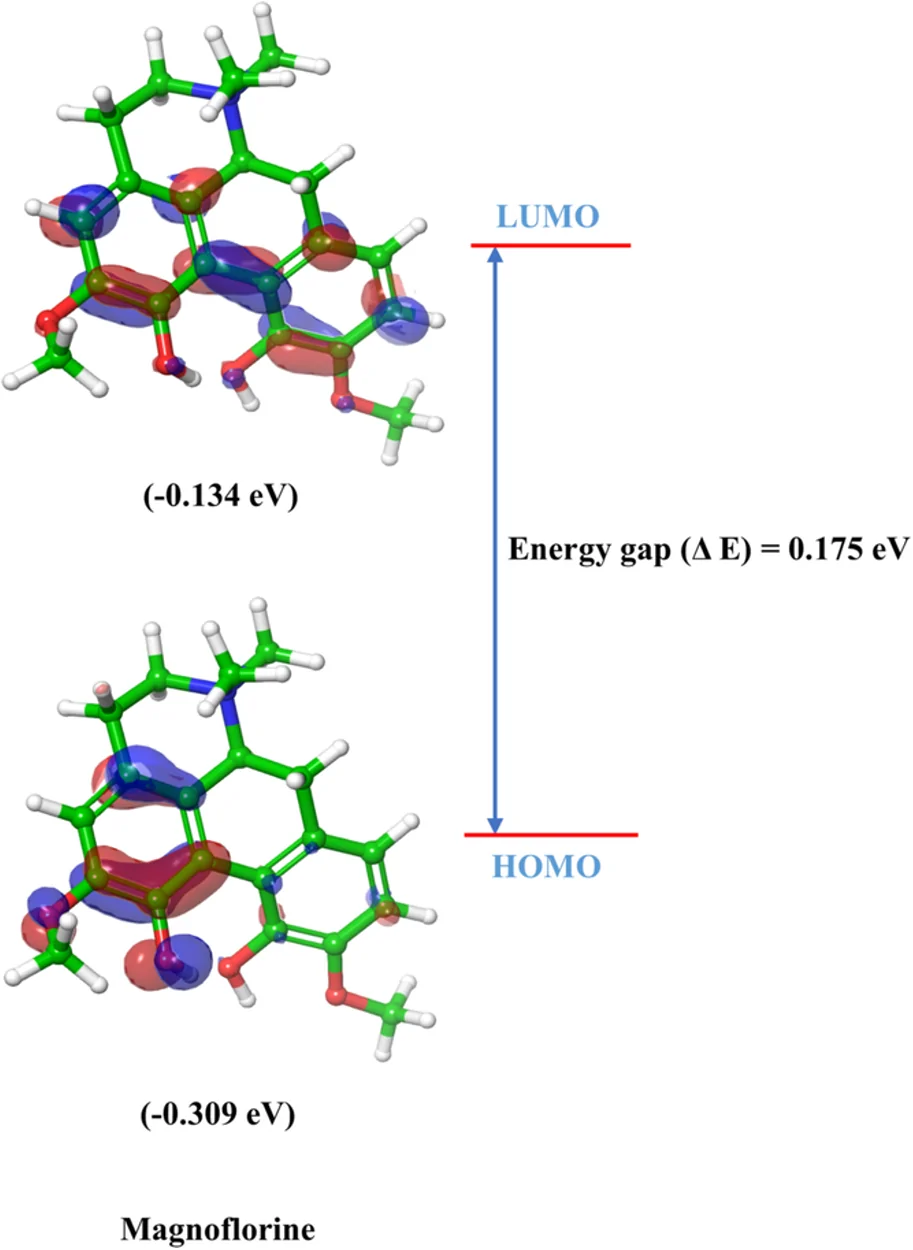
DFT‐optimized geometry and frontier molecular orbitals of Magnoflorine. Optimized molecular structure of Magnoflorine with spatial distribution and energy levels of the frontier molecular orbitals such as the Highest Occupied Molecular Orbital (HOMO) and Lowest Unoccupied Molecular Orbital (LUMO), with the HOMO‐LUMO energy gap (ΔE) computed at the B3LYP/6‐31 G level. The calculated ΔE is 0.175 eV.

### 3.4 MD Simulations of Caspase‐3‐Magnoflorine and Caspase‐3‐Kainic Acid Complexes

Caspase‐3‐Magnoflorine and Caspase‐3‐Kainic acid complexes were subjected to MD simulations for 200 ns to determine their thermodynamic stability, atomic‐level behavior, and binding pattern under physiological conditions with the target protein, i.e., Caspase‐3.

#### 3.4.1 Root Mean Square Deviation (RMSD)

The conformation of the complexes, that is, Caspase‐3‐Magnoflorine and Caspase‐3‐Kainic acid, was analyzed using RMSD, which determined the differences between the structures during the simulation period and their reference structure. The RMSD plot (as shown in Figure 1C) of the Caspase‐3‐Magnoflorine complex indicates that during the first 0–12 ns of the MD trajectory, there were notable fluctuations with RMSD values ranging between 0.4 and 1.34 nm. This initial fluctuation suggests conformational adjustments as the system responds to the binding of magnoflorine. Similar behavior has been observed for the initial phase of the Caspase‐3‐Kainic acid complex simulation, with a shorter time required for the conformational adjustments, which made the graph fluctuate in the first 15 ns, then started to stabilize to values close to 1 nm until the end of the first 100 ns of simulation. The Caspase‐3‐Magnoflorine complex took more time to stabilize, and fluctuations began to decrease only after 50 ns, even though the RMSD values were still higher than those observed in the Caspase‐3‐Kainic acid complex, which shows that the stability of Kainic acid with Caspase‐3 was higher for the first 100 ns. For the second 100 ns, the situation started to change for the Caspase‐3‐Kainic acid complex, as the frequency of fluctuations began to increase. The RMSD values were also higher, ranging between 1.01 and 1.26 nm. The situation was the opposite for the Caspase‐3‐Magnoflorine complex; the frequency of fluctuations decreased after 50 ns and remained at its lowest values till the end of the simulation (up to 200 ns), with less amount of fluctuations in RMSD values ranging between (1.13–1.23) nm. The reduced fluctuation in RMSD values suggests that the complex reached a more stable state. These observations suggest two phases of interaction; in the first phase (12–100 ns), the interaction between Kainic acid and Caspase‐3 was more stable, while in the second phase (60–200 ns), the interaction between Magnoflorine and Caspase‐3 was more stable. This supports the hypothesis that the binding between ligand and protein requires an initial period (0–12 ns) for the system to stabilize. The trend towards lower RMSD values over time aligns with the binding affinity results obtained from molecular docking and MMGBSA analysis, which indicated a more stable binding of Magnoflorine than Kainic acid with Caspase‐3.

#### 3.4.2 Radius of Gyration (rGy)

The radius of gyration (rGy) is useful for evaluating the size, shape, and compactness of biomolecular structures, such as proteins. It is derived by calculating the root mean square distance of all atoms from the center of mass of the structure. The rGy values of both complexes (as shown in Figure 1D) initially fluctuated between 1.5 and 1.9 nm for Magnoflorine and Kainic acid for 12 ns. Then, it remained stable at 1.4 nm for both complexes throughout the remaining simulation. The observed stabilization and reduction in the rGy value suggest that the Caspase‐3‐Magnoflorine and Caspase‐3‐Kainic acid complexes achieved compact and stable conformations, reflecting effective binding between Caspase‐3 and both ligands (Magnoflorine and Kainic acid).

#### 3.4.3 Root Mean Square Fluctuation (RMSF)

RMSFs were calculated to investigate the changes in Caspase‐3 residues due to its binding with Magnoflorine and Kainic acid. The RMSF values, as shown in the plot (Figure 1E), showed that only the Caspase‐3‐Magnoflorine complex exceeded 1 nm at the beginning of chain B, while the Caspase‐3‐Kainic acid complex did not exceed 1 nm at all. For both complexes, the RMSF value remained at 0.5 nm or below for the rest of the simulation. We have observed that most of the edges in the RMSF plot were identical or very similar for both complexes. This fluctuation between 0.1 and 0.5 nm indicates a high degree of overall stability in both complexes. This low RMSF value suggests that the complexes remain stable with only localized fluctuations in specific regions. Similarly, the RMSF value for Magnoflorine and Kainic acid was found to be between 0.01 and 0.14 nm (as shown in Figure 1F). The lower value of RMSF for both ligands suggests that both have been stabilized inside the binding pocket of the protein.

Overall, the MD simulation results corroborate the strong interaction and binding affinity between Caspase‐3 and Magnoflorine and Caspase‐3 and Kainic acid, and these outputs align with molecular docking results. The complexes undergo initial conformational adjustments, achieving a stable and compact state over time. These findings provide a comprehensive understanding of the dynamic behavior and stability of the Caspase‐3‐Magnoflorine and Caspase‐3‐Kainic acid complexes.

### 3.5 Neuroprotective Effect of Magnoflorine by Attenuating Kainic Acid‐Induced Reduction in Cell Viability

The MTT reduction assay was performed to assess the neuroprotective effect of Magnoflorine against the Kainic acid‐induced neurodegeneration and cell death. Magnoflorine showed neuroprotection by restoring the viability of retinoic acid‐differentiated SH‐SY5Y cells damaged by Kainic acid. 300 µM of Kainic acid reduced cell viability to approximately 46.85% of the control, which was significantly reversed to 85.23% by 20 µM of Magnoflorine, as shown in Figure 3.

**Figure 3:**
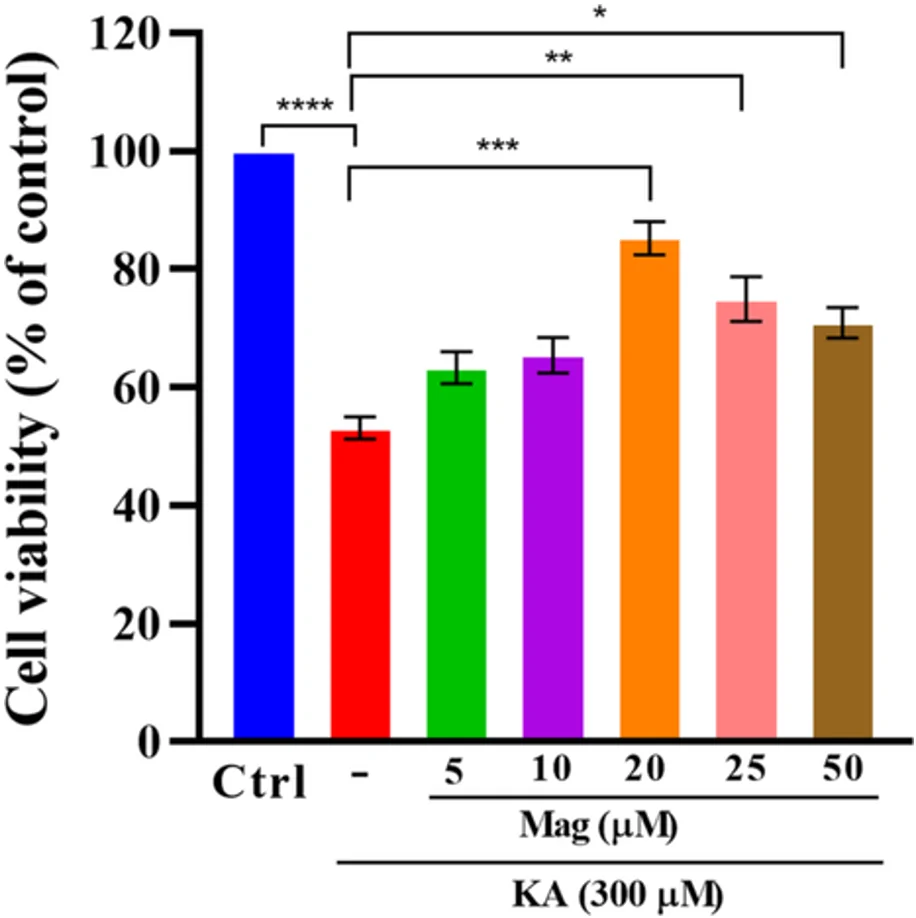
Neuroprotective effect of Magnoflorine against Kainic acid‐induced cell death. Cell viability was evaluated by MTT assay, in which retinoic acid‐differentiated SH‐SY5Y cells were pretreated in triplicate (indicative of three technical replicates) with various concentrations of Magnoflorine (5, 10, 20, 25, and 50 µM) for 24 h, followed by treatment with 300 µM Kainic acid (KA) for 24 h. ****p < 0.0001, ***p < 0.001, **p < 0.01, and *p < 0.05 were considered significant, and data are presented as the mean ± SEM from three independent experiments, indicating three biological replicates.

### 3.6 Neuroprotective Effect of Magnoflorine by Attenuating Kainic Acid‐Induced LDH Release

Magnoflorine also demonstrated neuroprotection in retinoic acid‐differentiated SH‐SY5Y cells damaged by Kainic acid by alleviating their LDH release. Kainic acid at 300 µM caused significant damage to the cell membrane and induced the release of LDH to 286.2% of control, while the pretreatment with Magnoflorine at 20 µM significantly reduced LDH release to approximately 132.9%, as shown in Figure 4.

**Figure 4:**
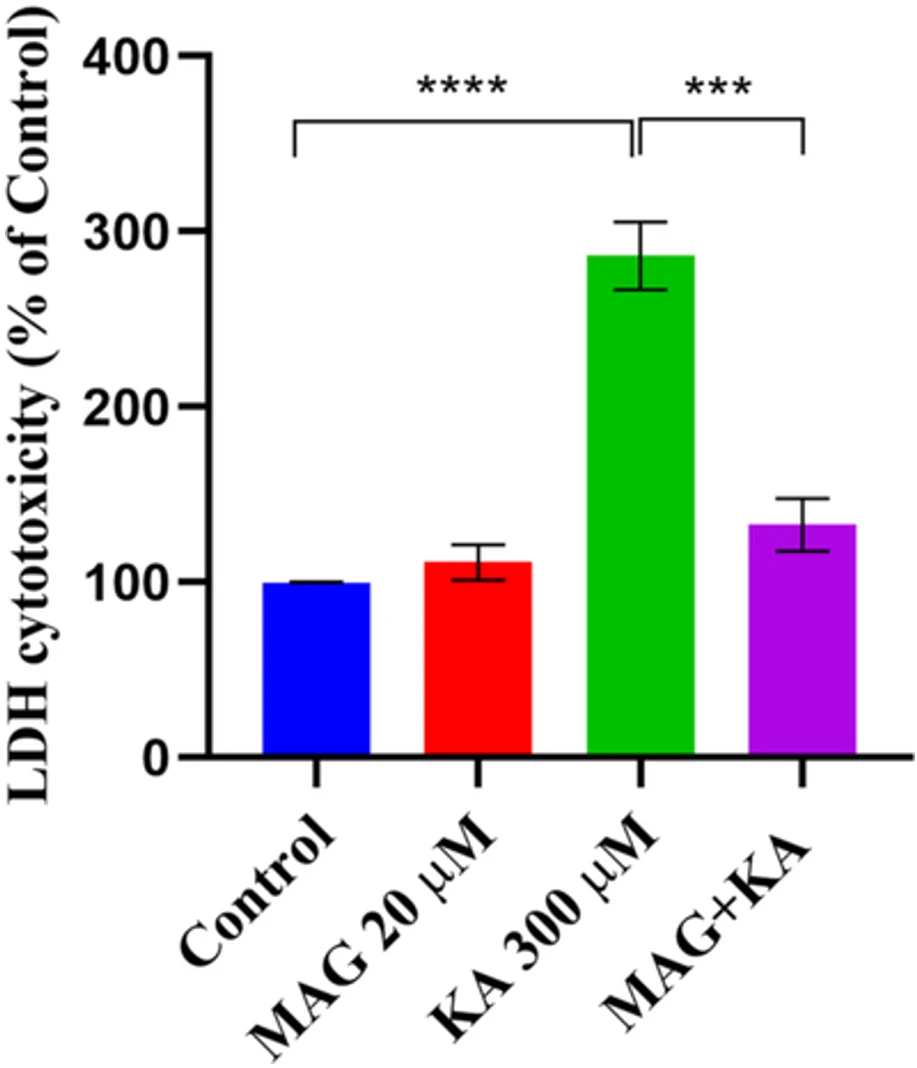
Neuroprotective effect of Magnoflorine against Kainic acid‐induced cytotoxicity. The LDH cytotoxicity assay determined cell cytotoxicity, where retinoic acid‐differentiated SH‐SY5Y cells were pre‐treated in triplicate (indicative of three technical replicates) with 20 µM Magnoflorine for 24 h, followed by treatment with 300 µM Kainic acid (KA) for the next 24 h. ****p < 0.0001 and ***p < 0.001 were considered significant, and data are presented as the mean ± SEM from three independent experiments, indicating three biological replicates.

### 3.7 Neuroprotective Effect of Magnoflorine by Reducing the Kainic Acid‐Induced Apoptosis

The Annexin V/PI assay was performed to evaluate the anti‐apoptotic effect of magnoflorine against Kainic acid‐induced apoptosis of retinoic acid‐differentiated SH‐SY5Y cells, which corresponds to its neuroprotective effect (as shown in Figure 5A,B). The lower left quadrant represents live or viable cells (Annexin V − /PI − ), the lower right quadrant denotes the early apoptotic cells (Annexin V + /PI − ), the upper right quadrant is for late apoptotic cells (Annexin V + /PI + ), while the upper left quadrant represents cells damaged during the whole procedure, i.e necrotic or dead cells (Annexin V − /PI + ). It has been found that Kainic acid significantly induced apoptosis (early 29.8% and late 34.9%), while magnoflorine showed neuroprotection and significantly alleviated Kainic acid‐induced cell death by decreasing early apoptosis to 13.4% and late apoptosis to 3.1%. This clearly demonstrates the anti‐apoptotic property of magnoflorine.

**Figure 5:**
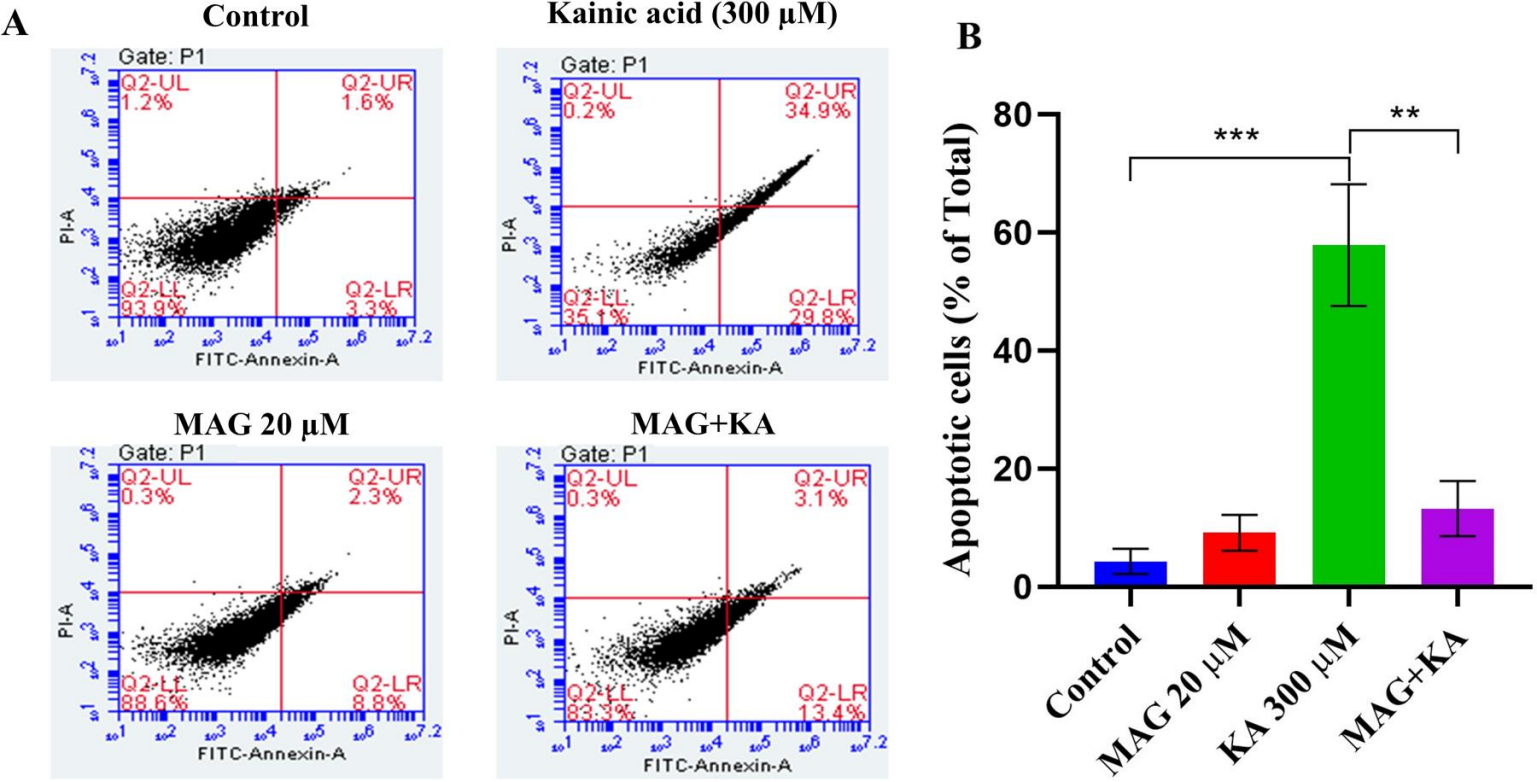
Quantitative analysis of apoptotic cells using Annexin V‐FITC/PI double staining and flow cytometry calculations. Annexin V/PI apoptosis assay was performed on retinoic acid‐differentiated SH‐SY5Y cells pre‐treated in triplicate (indicative of three technical replicates) with 20 µM Magnoflorine for 24 h, followed by treatment with 300 µM Kainic acid (KA) for the next 24 h to evaluate the anti‐apoptotic effect of magnoflorine against Kainic acid‐induced apoptosis. (A) Representative flow cytometry dot plots showing live cells in the lower‐left quadrant (Q2‐LL), early apoptotic cells in the lower‐right quadrant (Q2‐LR), late apoptotic cells in the upper‐right quadrant (Q2‐UR), and dead or necrotic cells in the upper‐left quadrant (Q2‐UL). (B) Quantitative analysis representing the percentage of total apoptotic cells (calculated as the sum of early and late apoptotic cells, Q2‐LR + Q2‐UR). ***p < 0.001 and **p < 0.01 were considered significant, and data are presented as the mean ± SEM from three independent experiments, indicating three biological replicates.

### 3.8 Neuroprotective Effect of Magnoflorine by Modulating the Kainic Acid‐Induced Upregulation of Caspase‐3 Expression

Immunocytochemical analysis of Caspase‐3 expression in retinoic acid‐differentiated SH‐SY5Y cells also highlighted the neuroprotective effect of magnoflorine. Magnoflorine significantly reduced the Kainic acid‐induced enhancement in the expression of Caspase‐3, as indicated by the mean fluorescence intensity. Treatment with kainic acid caused the upregulation of the expression of the pro‐apoptotic protein Caspase‐3, which was significantly reversed by Magnoflorine (as shown in Figure 6A,B).

**Figure 6:**
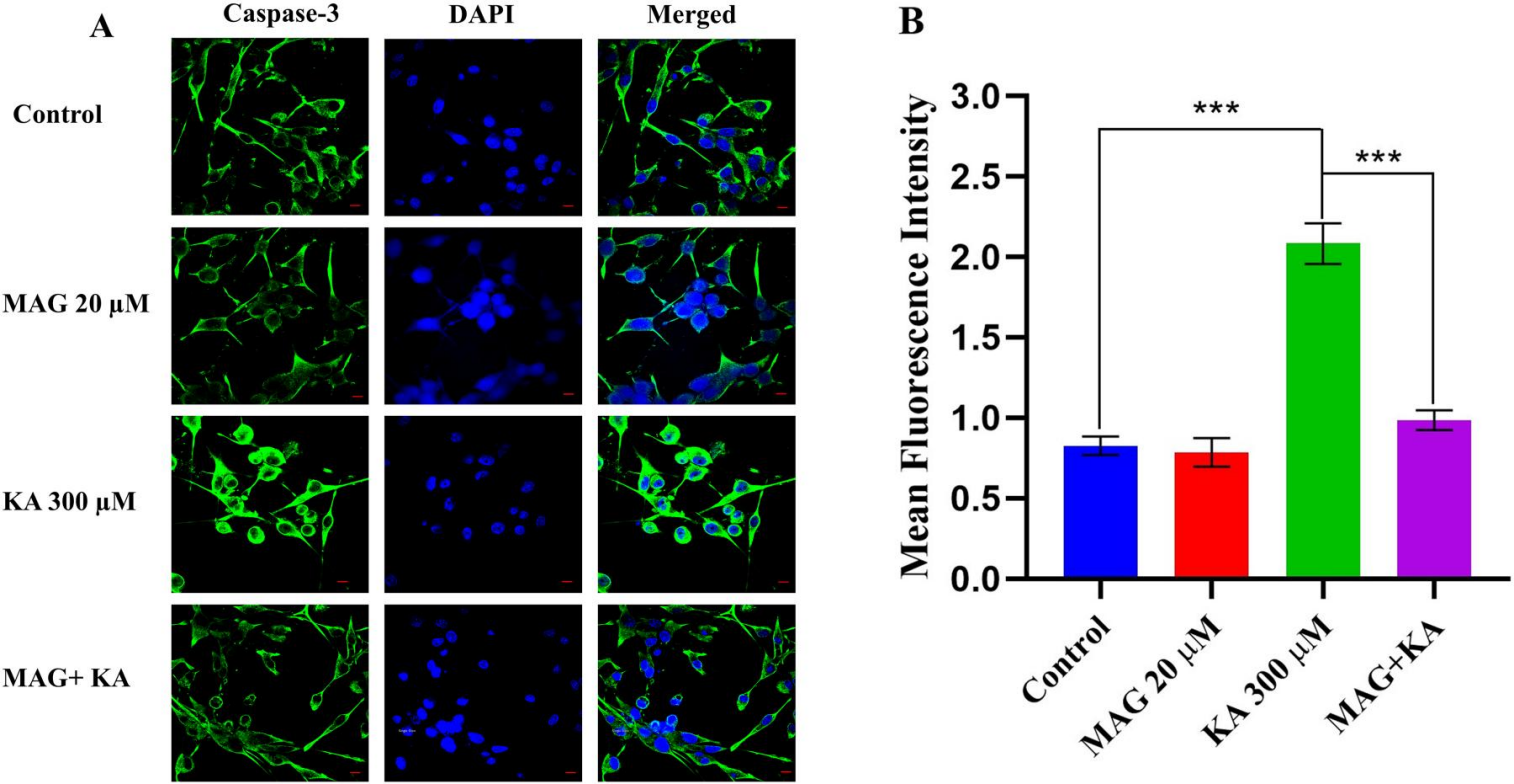
Quantitative confocal analysis of the neuroprotective effect of Magnoflorine against Kainic acid‐induced overexpression of caspase‐3. (A) Laser scanning confocal microscopy images (Scale bar 20 µm) showing caspase‐3 expression (green) in retinoic acid‐differentiated SH‐SY5Y cells pre‐treated in triplicate (indicative of three technical replicates) with 20 µM Magnoflorine, followed by treatment with 300 µM Kainic acid (KA). Nuclei are counterstained with DAPI (blue). (B) Quantification of mean fluorescence intensity of caspase‐3 across experimental groups. Statistical analysis of mean fluorescence intensity demonstrates a significant reduction in caspase‐3 expression upon magnoflorine co‐treatment. *** represents *p* < 0.001 and is considered significant. Data are presented as the mean ± SEM from three independent experiments, indicating three biological replicates.

## 4 Discussion

Medicinal plants are a great gift of nature to humanity because they produce a variety of bioactive secondary metabolites, which act as therapeutic agents and have many pharmacological activities. These metabolites are categorized into flavonoids, alkaloids, terpenoids, oils, phenolics, and waxes. Multiple in vitro and in vivo studies are required to assess the beneficial properties of these metabolites or phytochemicals. Due to advancements in technology, highly efficient softwares provides initial confirmation of the existence of medicinal properties in these compounds, which reduces the need for expensive and time‐consuming biological experiments. In our study, we emphasized the neurodegeneration induced by Kainic acid (a well‐known model of Epilepsy), and since neurodegeneration is associated with neuronal apoptosis, the involvement of activation of proteins like Caspase‐3 is evident [13, 16]. Our in silico analyses revealed that phytochemicals like Magnoflorine bind preferably and stably to the active sites of Caspase‐3 in the presence of Kainic acid, raising the possibility of their attenuating effect on neurodegeneration by inhibiting Caspase‐3 activation and maintaining its basal expression [35]. Magnoflorine binds preferably and more strongly to Caspase‐3 compared to Kainic acid during molecular docking. During MD simulations, Kainic acid was more stably bound than Magnoflorine to Caspase‐3 during the initial phase (12–100 ns) of RMSD, which aligns with the Kainic acid‐induced upregulation of Caspase‐3. Magnoflorine, however, binds stably and remains in the binding pocket of caspase‐3 during the longer phase (60–200 ns) of RMSD, in which Kainic acid became unstable, raising the possibility of its inhibition and reducing apoptosis. DFT analysis also revealed the highly reactive nature of Magnoflorine, which coincides with its observed biological activity. These observations were validated through in vitro analyses, where Magnoflorine provided neuroprotection by significantly reducing the Kainic acid‐induced death of retinoic acid‐differentiated SH‐SY5Y cells, which closely resemble mature human neurons. The neuroprotective effect of Magnoflorine has also been proven from the enhancement in the viability of these differentiated SH‐SY5Y cells by inhibiting their LDH release induced by Kainic acid. Annexin V/PI flow cytometry analysis demonstrated the neuroprotective effect of Magnoflorine through its anti‐apoptotic nature against the Kainic acid‐induced apoptosis of retinoic acid‐differentiated SH‐SY5Y cells. This effect was also observed during immunocytochemical analysis by analyzing the mean fluorescence intensity of Caspase‐3, which was observed to be significantly decreased by Magnoflorine. These observations clearly indicate that Magnoflorine confers neuroprotection by inhibiting the Kainic acid‐induced overexpression of Caspase‐3 and limiting neuronal apoptosis. Magnoflorine is an important quaternary aporphine alkaloid that has attracted extensive attention from researchers due to its various pharmacological activities, including anti‐inflammatory, antidiabetic, neuropsychopharmacological, antifungal, and antioxidant activities [10, 36, 37]. Magnoflorine is also observed to correct mitochondrial membrane potential and reduce reactive oxygen species (ROS), thereby inhibiting apoptosis. Magnoflorine further improved cognitive deficits and AD pathology by inhibiting the c‐Jun N‐terminal kinase (JNK) signaling pathway, highlighting its potential to be a therapeutic candidate for AD [38]. It has been reported to inhibit lipopolysaccharide‐induced apoptosis in murine macrophage cells [39]. Moreover, Magnoflorine can penetrate the blood‐brain barrier (BBB) and has shown marked central activity in mice that aligns with its observed drug‐likeness [40].

In a nutshell, we found that Magnoflorine has the potential to become a therapeutic candidate against various neurodegenerative diseases, as substantially demonstrated by previous studies as well as our various in silico analyses. Magnoflorine also significantly reduced the Kainic acid‐induced overexpression of caspase‐3, neurotoxicity, and neuronal apoptosis in vitro, which validated our in silico analyses. However, our study has certain limitations that should be acknowledged. First, the in vitro analyses using only retinoic acid‐differentiated SH‐SY5Y cells may not fully recapitulate all aspects of neurodegeneration pathology, one of which is neuroinflammation that involves microglial and astrocytic activation. Second, direct enzymatic inhibition assays for caspase‐3 were not performed. Moreover, the lack of BBB permeability validation due to the in vitro nature of our study warrants that proper in vivo studies are required to further validate the BBB permeability and neuroprotective potential of Magnoflorine against Kainic acid‐induced epileptic models and other epilepsy models.

## 5 Conclusion

Our study demonstrated the neuroprotective potential of the widely recognized phytochemical Magnoflorine through its anti‐apoptotic effect by inhibiting Kainic acid‐induced overexpression of Caspase‐3, a mediator of neurodegeneration and neuronal apoptosis. The integrated approach involving in silico and in vitro techniques provided significant evidence of the neuroprotective action of Magnoflorine against Kainic acid‐induced neuronal apoptosis. Molecular docking and MD simulations provided a basis for predicting stronger molecular interactions between Magnoflorine and Caspase‐3 than between Kainic acid and Caspase‐3, which indicates the Caspase‐3‐inhibiting nature of Magnoflorine. The in vitro analyses, including cell viability, cytotoxicity, and apoptosis assays, as well as immunocytochemistry, support our in silico findings by showing that Magnoflorine can reduce the Kainic acid‐induced apoptosis in retinoic acid‐differentiated SH‐SY5Y cells. These observations suggest that Magnoflorine may exert neuroprotection by attenuating neuronal apoptosis through the inhibition of Caspase‐3 signaling pathway, which still requires validation in the in vivo systems.

## Author Contributions

**Randeep Singh:** conceptualization, data curation, methodology, investigation, formal analysis, writing – original draft, writing – review and editing, visualization. **Sahil Arora:** methodology, data curation, software, formal analysis, writing – original draft, visualization. **Rimple Kaur:** methodology, formal analysis. **Ali Gharib Gharib:** methodology, data curation, software, formal analysis, writing – original draft, visualization. **Vikash Prashar:** writing – review and editing. **Tania Arora:** writing – review and editing. **Arti Sharma:** supervision, writing – review and editing, resources, validation. **Balachandar Vellingiri:** writing – review and editing. **Jyoti Parkash:** supervision, project administration, funding acquisition, resources, writing – review and editing, validation.

## Conflicts of Interest

The authors declare no conflicts of interest.

## Supporting information


Supporting File

## Data Availability

The data that support the findings of this study are available from the corresponding author upon reasonable request.
