# Supplementary material for: Neuroprotective Effect of Magnoflorine Through Inhibition of Caspase‐3‐Mediated Neuronal Apoptosis: In Silico to In Vitro Validation
Source: J Biochem Mol Toxicol. 2026 Nov 7;40(11):e71171. doi: 10.1002/jbt.71171 (PMC13744388; doi:10.1002/jbt.71171)
Supplement: Supplementary file 1 [file JBT-40-e71171-s001.docx]

**Calculation of the IC_50_ value of Kainic acid**

The IC_50_ value for Kainic acid was calculated using an MTT assay in a retinoic acid-differentiated SH-SY5Y cell line, as shown in **Figure S1**. SH-SY5Y cells were seeded at a density of 10^4^ cells/well in 96-well plates and differentiated with 10 µM retinoic acid solution. Differentiated cells were treated with a geometric or logarithmic range of concentrations of Kainic acid (12.5, 25, 50, 100, 200, 400, and 800 µM) prepared in DMEM for 24 h. The Kainic acid-treated medium was then replaced with 0.5 mg/ml MTT solution prepared in DMEM, and the plates were incubated for 3 h at 37°C in a humidified incubator containing 5% CO_2_. The formazan crystals formed in the wells were dissolved in DMSO, and the absorbance was read at 570 nm with a microplate reader.

**Figure S1. Kainic acid reduces the viability of retinoic acid-differentiated SH-SY5Y cells in a dose-dependent manner.** Retinoic acid-differentiated SH-SY5Y cells were treated with a geometric range of concentrations of Kainic acid (12.5-800 µM) for 24 h, and cell viability was evaluated using the MTT assay. Data points are expressed as a percentage of the untreated control (100% viability). Non-linear regression analysis was performed using a four-parameter logistic curve fit, with the bottom plateau constrained to 0% viability. The calculated IC₅₀ value is 345.4 µM (95% CI: 292.3 to 413.9 µM). Data represent the mean ± SD of three independent experiments, each performed in triplicate.

**Molecular docking of M826 with Caspase-3 (PDB ID: 3KJF)**

**Figure S2. Molecular docking interactions of Magnoflorine and the reference caspase-3 inhibitor M826 with caspase-3 (PDB ID: 3KJF).** The figure shows the docked binding poses and molecular interactions of (A) Magnoflorine and (B) M826 within the caspase-3 binding site. The interacting amino acid residues and relevant ligand-protein interactions (dashed lines) are indicated to compare their predicted binding modes.

**Confocal microscopy images for immunocytochemical analysis**

Given below are the unprocessed and uncropped confocal microscopy images taken at 60X magnification for each treatment group in the immunocytochemistry experiment.

Original image of Control

Original images of MAG 20 µM

Original image of KA 300 µM

Original image of MAG + KA

**Revalidated confocal microscopy images for immunocytochemical analysis**

Given below are the unprocessed and uncropped confocal microscopy images taken at 60X magnification for each treatment group in the immunocytochemistry experiment.

Original image of Control

Original image of MAG 20 µM

Original image of KA 300 µM

Original image of MAG + KA

**Figure S3. Quantitative confocal analysis of the neuroprotective effect of Magnoflorine against Kainic acid-induced overexpression of caspase-3.** Laser scanning confocal microscopy images showing caspase-3 expression (green) in retinoic acid-differentiated SH-SY5Y cells pre-treated in triplicate (indicative of three technical replicates) with 20 µM Magnoflorine, followed by treatment with 300 µM Kainic acid (KA). Nuclei are counterstained with DAPI (blue), and the mean fluorescence intensity of caspase-3 is measured across experimental groups. Statistical analysis of mean fluorescence intensity demonstrates a significant reduction in caspase-3 expression upon magnoflorine co-treatment. **** represents p<0.0001 and ** represents p<0.01 are considered significant. Data are presented as the mean ± SEM from three independent experiments, indicating three biological replicates.
